# Green (*Ulva fenestrata*) and Brown (*Saccharina latissima*) Macroalgae Similarly Modulate Inflammatory Signaling by Activating NF-*κ*B and Dampening IRF in Human Macrophage-Like Cells

**DOI:** 10.1155/2024/8121284

**Published:** 2024-05-17

**Authors:** Jennifer Mildenberger, Céline Rebours

**Affiliations:** Møreforsking AS, Borgundvegen 340, Ålesund 6009, Norway

## Abstract

Macroalgae are considered healthy food ingredients due to their content in numerous bioactive compounds, and the traditional use of whole macroalgae in Asian cuisine suggests a contribution to longevity. Although much information is available about the bioactivity of pure algal compounds, such as different polyphenols and polysaccharides, documentation of potential effects of whole macroalgae as part of Western diets is limited. Lifestyle- and age-related diseases, which have a high impact on population health, are closely connected to underlying chronic inflammation. Therefore, we have studied crude extracts of green (*Ulva fenestrata*) and brown (*Saccharina latissima*) macroalgae, as two of the most promising food macroalgae in the Nordic countries for their effect on inflammation *in vitro*. Human macrophage-like reporter THP-1 cells were treated with macroalgae extracts and stimulated with lipopolysaccharide (LPS) to induce inflammatory signalling. Effects of the macroalgae extracts were assessed on transcription factor activity of NF-*κ*B and IRF as well as secretion and/or expression of the cytokines TNF-*α* and IFN-*β* and chemokines IL-8 and CXCL10. The crude macroalgae extracts were further separated into polyphenol-enriched and polysaccharide-enriched fractions, which were also tested for their effect on transcription factor activity. Interestingly, we observed a selective activation of NF-*κ*B, when cells were treated with macroalgae extracts. On the other hand, pretreatment with macroalgae extracts selectively repressed IRF activation when inflammatory signaling was subsequently induced by LPS. This effect was consistent for both tested species as well as for polyphenol- and polysaccharide-enriched fractions, of which the latter had more pronounced effects. Overall, this is the first indication of how macroalgae could modulate inflammatory signaling by selective activation and subsequent repression of different pathways. Further *in vitro* and *in vivo* studies of this mechanism would be needed to understand how macroalgae consumption could influence the prevention of noncommunicable, lifestyle- and age-related diseases that are highly related to unbalanced inflammatory processes.

## 1. Introduction

Macroalgae are considered one of the alternative food sources for which the consumption is expected to augment worldwide. Its traditional inclusion in Asian cuisine suggests that regular macroalgae consumption might counteract the increasing incidence of lifestyle- and age-related diseases and contribute to Japanese longevity [1, 2]. In addition, there is an important interest in bioactive compounds from macroalgae with effects such as antioxidative, antimicrobial, or anti-inflammatory, which makes them interesting ingredients for functional food and cosmetical formulations [3, 4].

Dietary intake plays an important role in a functioning organism and overall performance. Much work has been done and is still underway to characterize and quantify potentially harmful elements in macroalgae such as iodine, arsenic, heavy metals, and marine allergens [5–7], as well as to reduce these compounds during processing [8, 9]. As the potential for safe inclusion in food products is more and more understood, it is of interest to better characterize and document the possible effects on health, when macroalgae are consumed as part of Western diets. Western diet is characterized by increased levels of saturated fat and refined sugar and is associated with a higher risk for metabolic, inflammatory, and age-related diseases, indicating that such an unfavorable diet contributes to morbidity [10, 11]. It is also acknowledged that noncommunicable diseases, such as atherosclerosis, cancer, Alzheimer's disease, diabetes type II, and many others, not only are associated with a certain lifestyle but also an underlying, low-grade chronic inflammation [12]. Nutritional changes, including an increased intake of macroalgae, could participate in the prevention or amelioration of age-related and noncommunicable diseases [13, 14]. In addition, much work is ongoing to identify drug leads from macroalgae compounds [15], which underlines their potential to provide a health benefit when introducing macroalgae as an alternative food source.

The main bioactive compounds in macroalgae are polysaccharides, phenolic compounds, carotenoids, vitamins, minerals, and peptides [4, 16, 17]. Fucoidans (sulphated polysaccharides) and phlorotannins (polyphenols) extracted from brown macroalgae have well-documented anti-inflammatory effects [18–21], but immune-stimulating effects have also been reported for fucoidan [22, 23]. Compared to brown macroalgae, the health effects of green macroalgae and their compounds are less studied. Still, green macroalgae are also reported to have anti-inflammatory as well as immunomodulatory effects [24]. Based on previous studies, it is clear that macroalgae influence inflammatory signaling, but it is difficult to predict the resulting benefit of anti-inflammatory or immune-stimulating effects of different species when consumed as part of human diets. There are many reasons why molecules might show opposing effects in different studies: different conditions of the biologic system (e.g., healthy or pathologic), impurities or combinations of compounds, different concentrations and incubation times, and also differently induced cellular inhibitors and/or activators.

Inflammation is important in the defence against pathogens but needs to be tightly regulated to prevent overwhelming (sepsis) or chronic inflammation. Thus, both anti-inflammatory and immune-stimulating effects might be beneficial under certain circumstances. On a molecular level, to mount an inflammation, pathogens or damage are sensed by cellular receptors, activating signaling pathways and leading to the secretion of inflammatory cytokines, which recruit immune cells to the insult. Nuclear factor-kappa B (NF-*κ*B) is a central transcription factor in inflammatory signaling and plays an important role in chronic diseases [25, 26]. It can be observed that most studies report results on markers induced by NF-*κ*B pathways. However, interferon regulatory factors (IRFs) are often simultaneously activated and contribute strongly to inflammatory signaling with the induction of interferon-regulated genes (ISGs) [27]. ISGs are not only crucial in antiviral defence but also play a role in the pathogenesis of autoimmune diseases [28, 29].

Much literature is available on the bioactivities of differently extracted fractions and compounds from macroalgae [3, 4]. Hence, when considering whole macroalgae as regular food ingredients, different compounds might have synergistic or anticipating effects. Therefore, we started with the assessment of a crude extract without further fractionation. *Saccharina latissima* is one of the most produced brown macroalgae in Europe [30], while *Ulva fenestrata* is a green macroalgae commonly found in Scandinavia. Both are of high interest for future use in food applications [22, 31, 32]. Our interest was also to study the effects of a green macroalgae besides the more extensively studied brown macroalgae. Therefore, as a first step to document potential health benefits for the inclusion of these two species in Western diets and to analyze their potential to counteract dysregulated inflammatory signaling, we have characterized the effect of crude extracts on both NF-*κ*B and IRF-driven pathways.

## 2. Materials and Methods

### 2.1. Macroalgae Culture

*S. latissima* biomass was produced in 2020 from integrated multitrophic aquaculture farms located at three sites in Norway (Bjønnspjotneset (S1), Furholmen (S2), Dyrholmen Vest, (S3)) owned, respectively, by Osland Havbruk AS, Sulefisk AS, and Engesund Fiskeoppdrett, localized in PO3 and PO4 production areas. Samples were collected as one bulk sample from several spots throughout each farm as described in Mildenberger, Stangeland, and Rebours, 2021 [6]. *U. fenestrata* (U) biomass was wild collected around the Island of Vigra (Norway). The algae were roughly washed right after collection and conserved at 12°C in seawater overnight. The biomass was then transported to the lab facilities of Møreforsking AS at Atlanterhavsparken, 6006 Ålesund, Norway, and was rinsed in three successive baths of 1 *µ*m filtrated and UV-treated seawater. The algae were maintained under standard culture conditions: 500 g of biomass in 500 L tanks with fluorescent light, air bubbling, 1 *µ*m filtrated, and UV-treated seawater supplemented with 10 mL of F/2 medium [23, 33] per 100 L seawater. Samples were harvested in three consecutive weeks (U1–U3).

### 2.2. Macroalgae Extracts Preparation

For all samples, the thalli were washed with seawater, drained from their surface water, frozen, and finally freeze-dried. Freeze-dried and ground macroalgae were extracted with phosphate-buffered saline (PBS) or dimethyl sulfoxide (DMSO) at 25 mg/mL in an ultrasonic water bath (Branson 2200, 40 kHz) for 20 min at room temperature. The ultrasonic water bath was used to limit viscosity and to increase the liberation of compounds. Samples were cooled, and filtered, and the crude liquid extract was used in experiments. As controls, the highest used concentrations of the buffer or solvent (PBS or DMSO) were added to control cells. Initial experiments were done with three repetitions with each *S. latissima* (S1–S3) or *U. fenestrata* sample (U1–U3), while subsequent experiments were done with either extract in different replicates and pooled in final results, indicated as U for *U. fenestrata* and S for *S. latissima*.

For the separation of crude polyphenols and polysaccharides, a simple extraction procedure was set up based on previous publications. In detail, 25 mg/mL dried macroalgae were incubated in 80% aqueous methanol for 30 min under rotation to extract polyphenols [34], then centrifuged at 3,000 *g* for 10 min to remove residual algae material. The supernatant was evaporated under nitrogen and redissolved in 1 mL PBS, representing the crude polyphenol fraction (PP). The pellet was further dissolved in 3 mL distilled water (dH_2_O) and incubated at 80°C for 2 hr, cooled, and precipitated with 4 : 1 ethanol at 4°C overnight [35–37]. The expected polysaccharides in this fraction were collected by centrifugation at 3,000 *g* for 10 min and the pellet dissolved in 1 mL PBS, representing the crude polysaccharide fraction (PS).

### 2.3. Total Phenolic Content (TPC)

TPC was measured by the Folin–Ciocalteu method [38, 39]. Gallic acid (Sigma–Aldrich G7384) standard was prepared in a range from 200 to 3.125 mg/L with two-times dilutions. In a microplate the following was mixed per well: 15 *µ*L sample, 170 *µ*L MilliQ-H_2_O, 12 *µ*L Folin–Ciocalteu reagent (2 N, Sigma–Aldrich 147641) and 30 *µ*L sodium carbonate (200 g/L). The plate was incubated in darkness for 1 hr, 73 *µ*L MilliQ-H_2_O was added, and absorption was read at 765 nm in a Synergy HTX S1LFA plate reader. Results for phenolic compounds were calculated based on a linear standard curve and are presented as gallic acid equivalents (GAE), with final values adjusted to the dry weight of the extracted samples.

### 2.4. Cell Culture

THP1-Dual™ NF-*κ*B-SEAP IRF-Luc Reporter monocytes (InvivoGen, thpd-nfis) were grown at a concentration of 0.5–2 mio cells/mL in RPMI 1640 (Thermofisher, 61870036) with 10% heat-inactivated fetal bovine serum (Sigma–Aldrich, F0804), 100 U/mL penicillin + 0.1 mg/mL streptomycin (Sigma–Aldrich, P4333), and 100 *μ*g/mL Normocin™ (InvivoGen, ant-nr-1) at 5% CO_2_ and 37°C, according to the manufacturer's instructions. To keep selection pressure, 10 *μ*g/mL Blasticidin (InvivoGen, ant-bl-1) and 100 *μ*g/mL Zeocin™ (InvivoGen, ant-zn-1) were added every second passage. For experiments, the cells were differentiated into macrophages by seeding with 40 ng/mL Phorbol 12-myristate 13-acetate (PMA; Sigma–Aldrich, P8139) and incubation for 3 days, followed by 1.5 days rest in fresh medium without PMA. To induce inflammatory signaling, cells were treated with 10 ng/mL lipopolysaccharide (LPS, Sigma–Aldrich, L2630).

### 2.5. Cellular Viability

Effects on cellular viability were analyzed by a resazurin-based assay (PrestoBlue™; Thermofisher, A13261) and spectrophotometric detection on a Synergy HTX S1LFA plate reader (BioTek) according to the manufacturer's instructions.

### 2.6. Reporter Assays

NF-*κ*B or IRF activation was analyzed by secreted embryonic alkaline phosphatase (SEAP) (QUANTI-Blue™, InvivoGen, rep-qbs) or Lucia luciferase (QUANTI-Luc™, InvivoGen, rep-qlc1) reporter assay in cell supernatants after stimulation with 10 ng/mL LPS for 24 hr, according to the manufacturer's instructions. QUANTI-Luc™ was analyzed immediately by luminescence and QUANTI-Blue™ after 45 min of incubation by absorbance at 635 nm on a Synergy HTX S1LFA plate reader (BioTek).

### 2.7. Enzyme-Linked Immunosorbent Assay (ELISA)

Cells were treated and stimulated by either LPS (10 ng/mL) or interferon-gamma (IFN-*γ*, 25 ng/mL) for 24 hr. Cytokine secretions were analyzed in technical duplicates in appropriately diluted medium from treated cells by Duo ELISA kits (R&D technologies) for human CXCL10 (dy266), IL-8 (dy208), and TNF-*α* (dy210) according to the manufacturer's instructions on a Synergy HTX S1LFA plate reader (BioTek). GainData® [40] was used with a four-parameter logistic regression standard curve for the calculation of cytokine concentrations.

### 2.8. Quantitative Real-Time PCR (qRT-PCR)

Cells were lysed and RNA was isolated by the Quick-RNA Miniprep Kit (Zymo Research, R1054). RNA concentration was measured by QuantiFluor® RNA System (Promega) on a Synergy HTX S1LFA plate reader (BioTek). cDNA was synthesized by the iScript cDNA kit (BioRad, 1708890) and run with the PrimePCR™ Probe Assays (BioRad) for human *TNF* (*qHsaCEP0040184*), *CXCL10* (*qHsaCEP0053880*), *IFNB1* (*qHsaCEP0054112*), *HPRT1* (*qHsaCIP0030549*), and *B2M* (*qHsaCIP0029872*) and the SsoAdvanced™ Universal Probes Supermix (BioRad, 1725280) on a CFX96 Real-Time PCR System (BioRad). The mean of *HPRT* and *B2M* Cts was used for normalization. Relative mRNA levels were transformed into a linear form by the 2 (−*ΔΔ*Ct) method [41]. Fold changes of RNA expression relative to unstimulated controls were used for statistical analysis.

### 2.9. Statistical Analysis

Statistical analyses were performed in GraphPad Prism 10.0.1. Ordinary one-way ANOVA was performed on data from at least three independent repetitions of all experiments. Dunnett's multiple comparisons test was run to compare all treated samples to the relevant control (the LPS-stimulated control in induced state and the untreated control in the basal state) and pairwise comparisons are shown with multiplicity adjusted p-value. For graphs with superscript letters to indicate similar means, ANOVA followed by Tukey post hoc analysis and allocation of groups was performed in R4.3.1/RStudio. Graphs present mean values with standard deviation and individual values are shown as points.

## 3. Results

### 3.1. Determination of the Tolerable Range of Macroalgae Treatments

**Figure 1:**
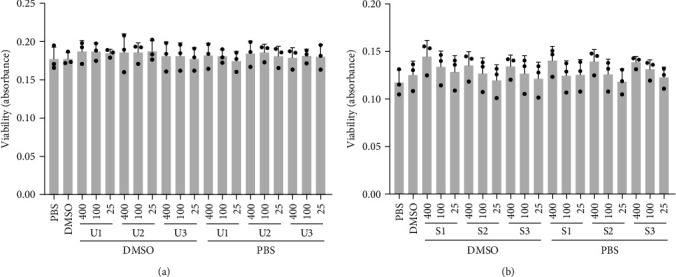
Viability of THP-1 macrophages treated with PBS or DMSO extracts of *U. fenestrata*, indicated as U1–U3 (a) or *S. latissima*, indicated as S1–S3 (b) at indicated concentrations (400, 100, and 25 *µ*g/mL) for 24 hr. Viability was assessed by a resazurin-based assay and mean absorbance with standard deviation and individual values are shown. All treatments were compared to the untreated controls (solely PBS or DMSO without macroalgae extract).

As a simplified model for dietary intake of whole macroalgae, we assessed the effects of crude extracts of green and brown macroalgae without further fractionation. Extracts of green (*U. fenestrata*) and brown macroalgae (*S. latissima*) were initially prepared in PBS as a physiological medium or DMSO to increase the solubility of certain hydrophobic bioactive compounds, such as polyphenols. To determine the tolerable range of macroalgae treatments, THP-1 macrophages were treated with macroalgae extracts in PBS or DMSO at 25–400 *µ*g/mL for 24 hr, and cell viability was assessed. The macroalgae treatments were well tolerated, and viability was not significantly different from control cells treated with, respectively, PBS or DMSO alone (Figure 1).

### 3.2. Influence of Macroalgae Extracts on NF-*κ*B and IRF Activation

**Figure 2:**
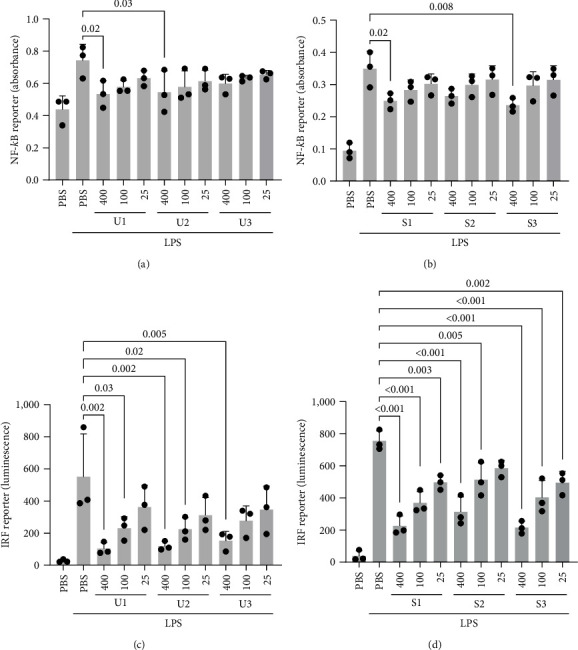
NF-*κ*B reporter activation in THP-1 macrophages pretreated with *U. fenestrata*, indicated as U1–U3 (a) or *S. latissima*, indicated as S1–S3 (b) extracted in PBS at indicated concentrations (400, 100, and 25 *µ*g/mL) for 16 hr, followed by LPS stimulation (10 ng/mL) for 24 hr. IRF reporter activation in THP-1 macrophages pretreated with *U. fenestrata* (c) or *S. latissima* (d) extracts in PBS at indicated concentrations (400, 100 and 25 *µ*g/mL) for 16 hr, followed by LPS stimulation (10 ng/mL) for 24 hr. All treatments were compared to the LPS-stimulated control.

Most lifestyle and age-associated chronic diseases have an underlying chronic inflammation driving their pathologic changes. In light of extensive reports on anti-inflammatory effects of macroalgae compounds, we analyzed the influence of macroalgae extracts on NF-*κ*B and IRF activation as two of the driving transcription factors in inflammatory signaling. Cells were pretreated with extracts of green and brown macroalgae for 16 hr and were stimulated with the bacterial cell wall component LPS for 24 hr to activate both inflammatory pathways, monitored by NF-*κ*B and IRF reporters. We observed a slightly lowering effect on NF-*κ*B activation by some of the green macroalgae *U. fenestrata* samples (Figures 2(a) and S1(A)), similarly to some of the brown macroalgae extracts of *S. latissima* (Figures 2(b) and S1(B)) when pretreated for 16 hr. On the other hand, treatment with both green and brown macroalgae had a pronounced limiting and concentration-dependent effect on IRF activity (Figures 2(c), 2(d), S1(C), and S1(D)). Pretreatment for 2 hr was tested with pooled *S. latissima* extracts and showed a significant but very marginal dampening effect on NF-*κ*B, while no effect was seen on IRF activity (Figures S2(A) and S2(B)). Thus, further experiments were done with 16 hr pretreatment. PBS (Figure 2) and DMSO extracts (Figure S1) performed similarly, despite the higher content of phenolic compounds in the DMSO extracts (Figures S2(E) and S2(F)). We selected the PBS extracts as the most relevant extracts for further experiments, being closer to dietary conditions. The three samples of *S. latissima*, harvested from different locations and the three *U. fenestrata* samples, collected in consecutive weeks, did not differ in their effects.

### 3.3. Effects of Macroalgae Extracts on Cytokine and Chemokine Secretion

**Figure 3:**
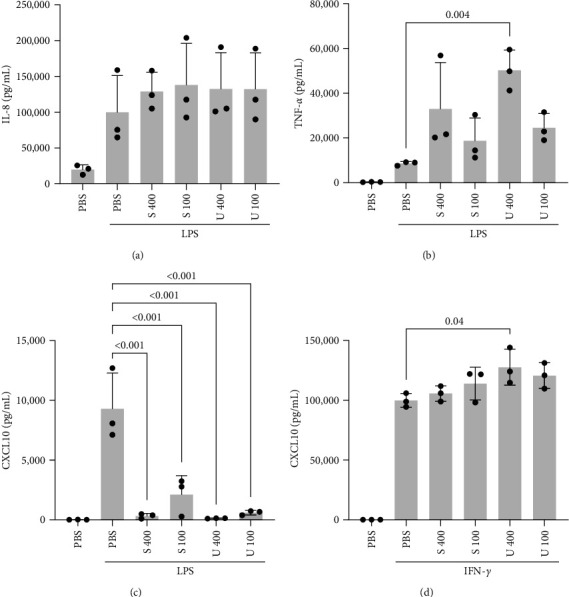
THP-1 macrophages were pretreated with *S. latissima* (indicated as S) or *U. fenestrata* (indicated as U) extracts in PBS at indicated concentrations (400 and 100 *µ*g/mL) for 16 hr, followed by stimulation with LPS (10 ng/mL) for 24 hr. IL-8 (a), TNF-*α* (b), and CXCL10 (c) secretion were assessed by ELISA. CXCL10 secretion was also assessed in identically pretreated, and IFN-*γ* (25 ng/mL) stimulated cells (d). All treatments were compared to the LPS-stimulated control.

To confirm the effect of NF-*κ*B and IRF activation on protein level, the secretion of chemokine IL-8 and cytokine TNF-*α* were assessed in cell supernatants as NF-*κ*B-regulated targets. No effect was observed on IL-8 secretion (Figure 3(a)) and TNF-*α* showed rather a significant increase for the highest concentration of *U. fenestrata* extract used (Figure 3(b)). CXCL10 is both an NF-*κ*B and IRF-dependent chemokine [42] and an important attractant of adaptive immune cells as well as driver of inflammation in many pathologic conditions [43]. CXCL10 showed a strong downregulation for all concentrations of both *U. fenestrata* and *S. latissima* extracts (Figure 3(c)). CXCL10 is also induced by interferons (IFN) and activation of the Janus kinase/signal transducer and activator of transcription (JAK/STAT) signaling pathway, either through feedback loops after activation of pattern-recognition receptors (PRR) or independent of the activation of NF-*κ*B and IRFs [44]. To assess the IFN-activated pathway, cells were stimulated by IFN-*γ* instead of LPS, but the macroalgae extracts did not reduce CXCL10 secretion induced by this pathway (Figure 3(d)).

### 3.4. Effects of Macroalgae Extracts on Expression of Cytokine and Chemokine mRNA

**Figure 4:**
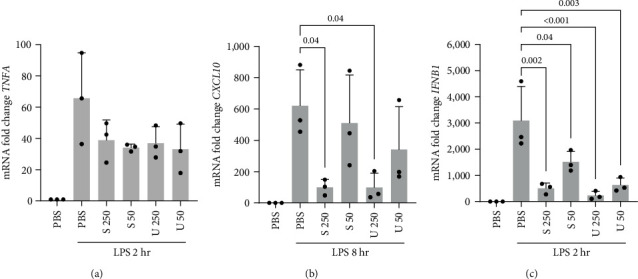
*TNFA* (a), *CXCL10* (b), and *IFNB1* (c) mRNA levels assessed by qrt-PCR in THP-1 macrophages pretreated with *S. latissima* (indicated as S) or *U. fenestrata* (indicated as U) extracts in PBS at indicated concentrations (250 and 50 *µ*g/mL) for 16 hr, followed by stimulation with LPS (10 ng/mL) for 2 or 8 hr, as indicated. Fold changes relative to untreated control are shown. All treatments were compared to the LPS-treated control.

The expression of NF-*κ*B and IRF target genes was also analyzed at the mRNA level. As the highest used concentrations of the extracts led to a nearly complete downregulation of CXCL10 on protein level, slightly lower concentrations were used for mRNA analyses. *TNFA* mRNA was assessed at 2 hr after LPS stimulation and *CXCL10* at 8 hr after LPS activation according to the previously observed induction peak (Figures S2(C) and S2(D)). No significant effect on *TNFA* mRNA expression was seen (Figure 4(a)). *CXCL10* mRNA expression was downregulated by treatment with 250 *µ*g/mL of macroalgae extracts, corresponding but less powerful than on protein level (Figure 4(b)). As IRF activation induces IFNs, which create a feedback loop and contribute to further expression of later target genes (such as *CXCL10*), we analyzed the expression of *IFNB1* after 2 hr of LPS stimulation. *IFNB1* was strongly downregulated by treatment with the macroalgae extracts, indicating that inhibition of this pathway occurs earlier than the *CXCL10* expression peak (Figure 4(c)).

### 3.5. Activation of NF-*κ*B by Macroalgae Extracts

**Figure 5:**
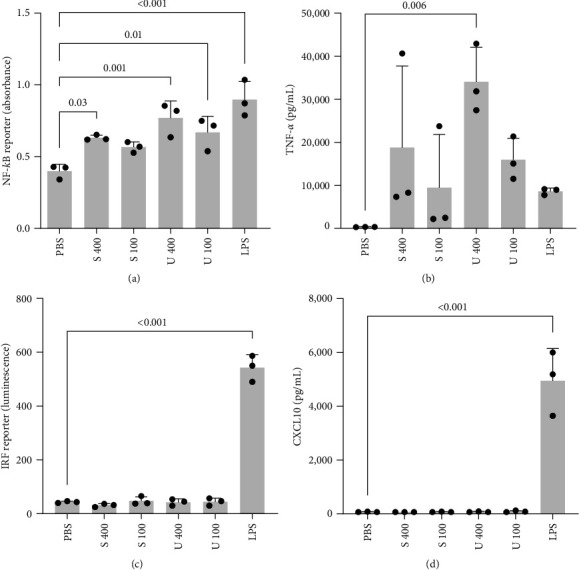
THP-1 macrophages were treated with *S. latissima* (S) or *U. fenestrata* (U) extracts alone at indicated concentrations (400 and 100 *µ*g/mL) or LPS (10 ng/mL) for 24 hr. NF-*κ*B reporter activation (a) and IRF reporter activation (c) were analyzed by their respective reporter assays. TNF-*α* (b) and CXCL10 (d) secretion were analyzed by ELISA. All treatments were compared to the PBS control.

Several studies report immune-stimulatory effects by polysaccharides of macroalgae [24, 45] and we observed an increased induction of TNF-*α* on protein level after treatment with *U. fenestrata* extracts and LPS stimulation (Figure 3(b)). Therefore, to analyze the effect of macroalgae extracts in unstimulated conditions, reporter cells were incubated with the macroalgae extracts alone for 16 hr, while LPS was included only as the control. Both brown and green macroalgae extracts significantly increased NF-*κ*B activation, even more pronounced for *U. fenestrata*, and similar to LPS (Figure 5(a)). A similar pattern was seen on protein level with a strong but more variable induction of TNF-*α*, only significantly different from control for the highest concentration of *U. fenestrata* extract (Figure 5(b)). None of the macroalgae treatments were able to activate IRF (Figure 5(c)) or induce CXCL10 secretion (Figure 5(d)).

### 3.6. Influence of Polyphenol- and Polysaccharide-Enriched Fractions on NF-*κ*B and IRF Activation

**Figure 6:**
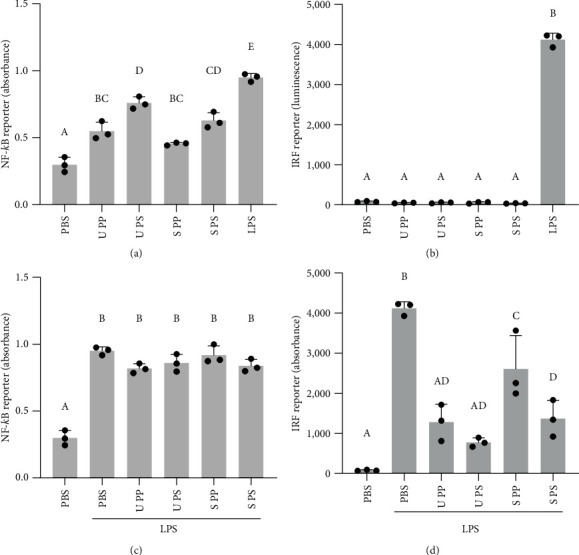
THP-1 macrophages were treated with polyphenol-enriched (PP) and polysaccharide-enriched (PS) fractions of *U. fenestrata* or *S. latissima* extracted from 800 *µ*g/mL whole dried seaweed for 16 hr and analyzed for NF-*κ*B (a) and IRF (b) reporter activation. In the same way, treated cells were further stimulated by LPS for 24 hr and analyzed for NF-*κ*B (c) and IRF (d) reporter activation. All treatments were compared to each other, and similar means are indicated by the same superscript letter.

Finally, to see if a basic fractionation of the extracts affects the observed immune-stimulatory and anti-inflammatory effects, we prepared fractions enriched in polyphenols (indicated as PP) or reduced in polyphenols and enriched in polysaccharides (indicated as PS) of both *U. fenestrata* and *S. latissima*. The content of total phenolic compounds was strongly reduced in the PS fractions (Figures S2(E) and S2(F)). Both PP and PS fractions were able to activate NF-*κ*B similar to the whole extracts and with a stronger effect of the PS fraction (Figure 6(a)). No activation of IRF signaling was observed (Figure 6(b)). When cells were further stimulated by LPS, no major effect on NF-*κ*B signaling was seen (Figure 6(c)), whereas IRF signaling was significantly dampened by all fractions (Figure 6(d)), and for *S. latissima* samples stronger by the PS fraction.

**Figure 7:**
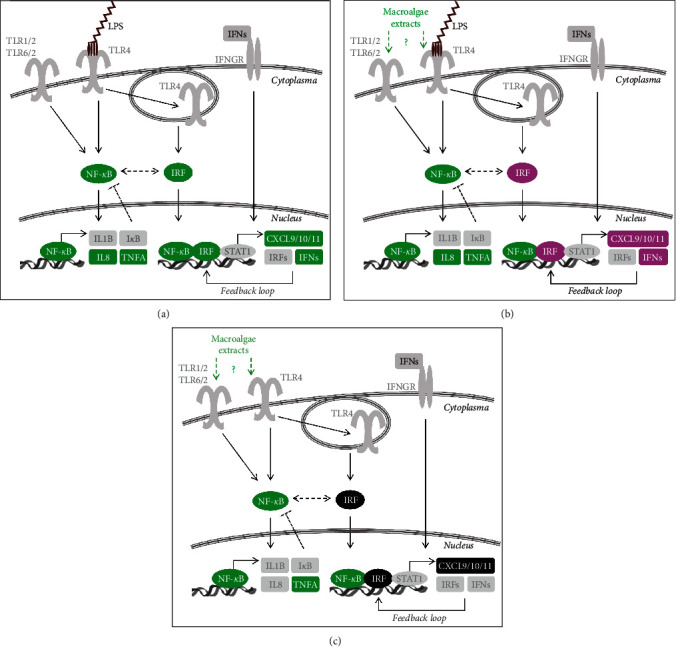
Schematic and simplified illustration of the analyzed inflammatory signaling pathways and observed effects by treatment with crude extracts of both brown (*S. latissima*) and green (*U. fenestrata*) macroalgae. (a) Signaling induced by LPS. Binding of LPS activates NF-*κ*B and internalization of the receptor (TLR4) activates IRF signaling pathways. (b) Selective inhibition of LPS-induced signaling with dampened IRF signaling in case of pretreatment with macroalgae extracts. (c) Induction of NF-*κ*B signaling, but not IRF signaling by macroalgae extracts alone. Tested mediators are colored green when activated, magenta when reduced, and black when unchanged.

Taken together, by treatment with different extracts of both green *U. fenestrata* and brown *S. latissima* macroalgae, NF-*κ*B signaling was induced in human macrophages. When pretreated with macroalgae extracts and stimulated by LPS, IRF signaling including expression of *IFNB1* and *CXCL10* was strongly reduced. Based on these preliminary results, we suggest a simplified scheme of the tested pathways and macroalgae-mediated effects (Figure 7).

## 4. Discussion

The ongoing development of alternative and sustainable food sources is a chance to introduce more healthy diets, in which macroalgae can play a role with their numerous bioactive compounds [3, 4]. However, resulting health effects are not easy to predict, thinking of whole macroalgae as a regular food ingredient with different compounds having synergistic or antagonistic effects. In previous studies, both polysaccharides and polyphenols from brown and green macroalgae were reported to have immunostimulatory or dampening effects in different models. For brown macroalgae, different fucoidan extracts have reduced the presence of inflammatory markers [46–48] and limited inflammasome activation in cells or mice [49]. Similar alleviating effects in inflammatory mouse models were seen with extracts rich in phlorotannins and other brown macroalgae polyphenols [50–52]. The carotenoid fucoxanthin has also shown antioxidant and anti-inflammatory effects in several studies [53, 54]. On the contrary, other studies demonstrated immune-stimulating effects for fucoidan [45, 55] and alginate oligosaccharides [56, 57]. For green macroalgae, the sulfated polysaccharide ulvan has shown immune-stimulating effects providing increased protection against infection when used as ingredients in aquafeed or husbandry animal feed [58–61]. In cell models treated with *Ulva* polysaccharides, an increase in inflammatory cytokines has been observed [62, 63]. It was further demonstrated that the effect on inflammatory signaling depends on the molecular weight of the ulvan molecule [64]. In contrast, a decrease in inflammatory markers was reported in a mouse model of breast cancer and in aged mice that consumed *Ulva lactuca* polysaccharides [65, 66].

We have characterized the effect of crude extracts of the green macroalgae *U. fenestrata* as well as of the brown kelp species *S. latissima* on different inflammatory pathways. Crude extracts from both macroalgae showed basal activation of NF-*κ*B and a strong reduction in IRF signaling when inflammatory signaling was induced. In light of many different and partly contradictive reports on macroalgae bioactivity, it seems surprising that a green macroalgae extract behaves so similarly to a brown macroalgae extract. However, many of these studies are done with purified compounds, and brown and green macroalgae are rarely compared in the same conditions. Olsthoorn et al. [20] published an extensive review on the effects of brown macroalgae on inflammation and conclude with four main concepts including an inhibitory effect on the acute phase of inflammation, which is seen across many different studies, extracts, and compounds [20]. In another study, the polyphenol-rich extracts of brown (*Laminaria japonica*), green (*U. lactuca*), and red macroalgae (*Porphyra tenera*) have shown comparable antioxidative capacity and lowering effects on nitric oxide production and *TNFA* expression when assessed in mouse macrophages [67]. We provide here an indication that immune-stimulating and anti-inflammatory effects might coexist, depending on which pathway is mainly affected and if effects are observed in a basal or induced cellular state.

The effects of brown macroalgae on inflammation have further been examined in a few human studies. Brown macroalgae supplements have been tested in the management and prevention of metabolic syndrome with promising results such as beneficial effects on insulin levels [68]. Nevertheless, further research is warranted [69]. Brown macroalgae polyphenols were shown to reduce TNF-*α* in dyslipidemic patients or prediabetic subjects [70] and limited the increase of TNF-*α* in overweight and obese prediabetic subjects [71]. Another study reported the effects of a polyphenol-rich extract from brown macroalgae on antioxidant activity and DNA damage in an obese population but without any change in the measured cytokines [72]. In future studies, it would be interesting to test the here made observations *in vivo* focusing on the selective activation or repression of NF-*κ*B and IRF pathways.

Water-based extracts are not expected to contain prominent amounts of polyphenols, which are normally extracted with organic solvents [73]. Also, Laminariales have a high content of fucoidans and carotenoids, while a rather low content in phenolic compounds or phlorotannins [74]. On the contrary, polysaccharides such as fucoidan and ulvan are readily water-soluble [75, 76], and expected to be present in the crude whole extracts used in this study. We show that initial DMSO extracts had a higher content in polyphenols than PBS extracts, while both showed similar effects on inflammation. Also, polyphenol-reduced/polysaccharide-enriched fractions (PS) performed rather better than polyphenol-enriched fractions (PP). Thus, polysaccharides are expected to play a role for the here-described effects. As this study did not further investigate polysaccharide content or structure, it will be further interesting to compare the here observed effects to the ones obtained with pure and identified polysaccharides. Both ulvan and fucoidan have been shown to activate inflammatory signaling [24, 45]. For ulvan, it has further been proposed that this effect is mediated through the PRRs toll-like receptor 2 (TLR2) and TLR4, where also LPS binds to initiate the inflammatory signaling cascade [56, 58, 59]. Activation of IRFs through PRRs depends on the endocytosis of the receptor [77, 78] and it would be interesting to see if binding of specific macroalgae polysaccharides to the receptor might inhibit this mechanism.

It is well-known that negative feedback loops keep inflammatory signaling in balance and numerous antagonists are induced together with active inflammatory signaling [79], suggesting that IRF repression could be the cause of precedent NF-*κ*B activation. NF-*κ*B and IRF signaling are highly connected [80]. NF-*κ*B is necessary for the induction of anti-inflammatory mediators and is equally important in inflammation and resolution [81, 82]. Many ISGs are induced by interferons and further restrict inflammatory signaling. Likewise, suppressors of cytokine signaling (SOC) are expressed downstream of the JAK/STAT axis and suppress TLR signaling [83, 84]. Finally, the induction of a refractory state after LPS stimulation has been described with the downregulation of many tolerizeable genes, including NF-*κ*B, TNF-*α*, or IFN-*β* [85, 86]. However, induction of a generally tolerant state after TLR activation by macroalgae polysaccharides would not explain the selectivity of subsequent IRF repression and it remains to determine if and how the presented findings are linked to each other.

We have observed a strong reduction of IRF signaling after pretreatment with macroalgae extracts. Importantly, both chronic inflammation and age-related pathologies have been linked to unbalanced IFN signaling [87–89]. Moreover, aging itself and most changes in aging tissues are accompanied by the induction of IFN-responsive genes [90–92]. Interestingly, cognitive function could be partly restored by blocking IFN-dependent signaling in the brains of aging mice [93]. Sayed et al. [94] found that the chemokine CXCL9 determines aging and although we only assessed CXCL10 here, likely, we could also have observed a repression of CXCL9 after treatment with macroalgae extracts. In this aspect, modulation of IRF signaling by macroalgae, and importantly by green and brown macroalgae, is highly interesting for further studies regarding their potential preventive effect on the development of age-related diseases in general.

## 5. Conclusions

Macroalgae are thought of as new healthy food alternatives. Here we have analyzed some aspects of the regulatory impact of green and brown macroalgae extracts on inflammation. We found a basal boosting effect on NF-*κ*B activation, while a dampening of IRF signaling in LPS-induced state. This is a highly interesting finding, keeping in mind the dysregulated inflammatory signaling that is a common driving factor in many noncommunicable and age-related diseases. There is a long way for these findings to be verified *in vivo*. Further studies will be needed to explicitly identify and characterize the active compounds in the here-described crude extracts for a better understanding of the presented bioactive effects. Closer examination (e.g. -*omics* of cellular responses, bioavailability, receptor interactions), as well as comparison of the observed effects to the performance of pure polysaccharides, could provide further valuable information about regulatory effects on inflammation that could have a beneficial effect on population health when including macroalgae in Western diets.

## Data Availability

All information to reproduce the presented analyses are included in the manuscript and no other new data were generated. Original material will not be sufficient for distribution.

## Abbreviations

B2M: Beta-2 microglobulin
CXCL10: C-X-C motif chemokine ligand 10
DMSO: Dimethyl sulfoxide
HPRT: Hypoxanthine Phosphoribosyltransferase 1
IFN: Interferon
IL: Interleukin
IRF: Interferon regulatory factor
ISGs: Interferon-stimulated genes
JAK: Janus kinase
LPS: Lipopolysaccharide A
NF-*κ*B: Nuclear factor-kappa B
PBS: Phosphate-buffered saline
PMA: Phorbol 12-myristate 13-acetate
PRR: Pattern recognition receptor
SEAP: Secreted embryonic alkaline phosphatase
SOC: Suppressors of cytokine signaling
STAT: Signal transducer and activator of transcription
TLR: Toll-like receptor
TNF-*α*: Tumor necrosis factor-alpha.

## Supplementary Materials

Supplementary Materials Figure S1: responses on transcription factor activity by treatment with DMSO extracts for 24 hr (Figure S1) or treatment with PBS and DMSO extracts for 2 hr (Figures S2(A) and S2(B)). Figure S2: induction peaks of TNFA and CXCL10 expression (Figures S2(B) and S2(C)). Total phenolic content in macroalgae samples (Figures S2(E) and S2(F)).
