# Supplementary material for: Green (Ulva fenestrata) and Brown (Saccharina latissima) Macroalgae Similarly Modulate Inflammatory Signaling by Activating NF-κB and Dampening IRF in Human Macrophage-Like Cells
Source: J Immunol Res. 2024 May 17;2024:8121284. doi: 10.1155/2024/8121284 (PMC11126347; doi:10.1155/2024/8121284)
Supplement: Supplementary Materials — Figure S1: responses on transcription factor activity by treatment with DMSO extracts for 24 hr (Figure S1) or treatment with PBS and DMSO extracts for 2 hr (Figures S2(A) and S2(B)). Figure S2: induction peaks of TNFA and CXCL10 expression (Figures S2(B) and S2(C)). Total phenolic content in macroalgae samples (Figures S2(E) and S2(F)). [file 8121284.f1.docx]

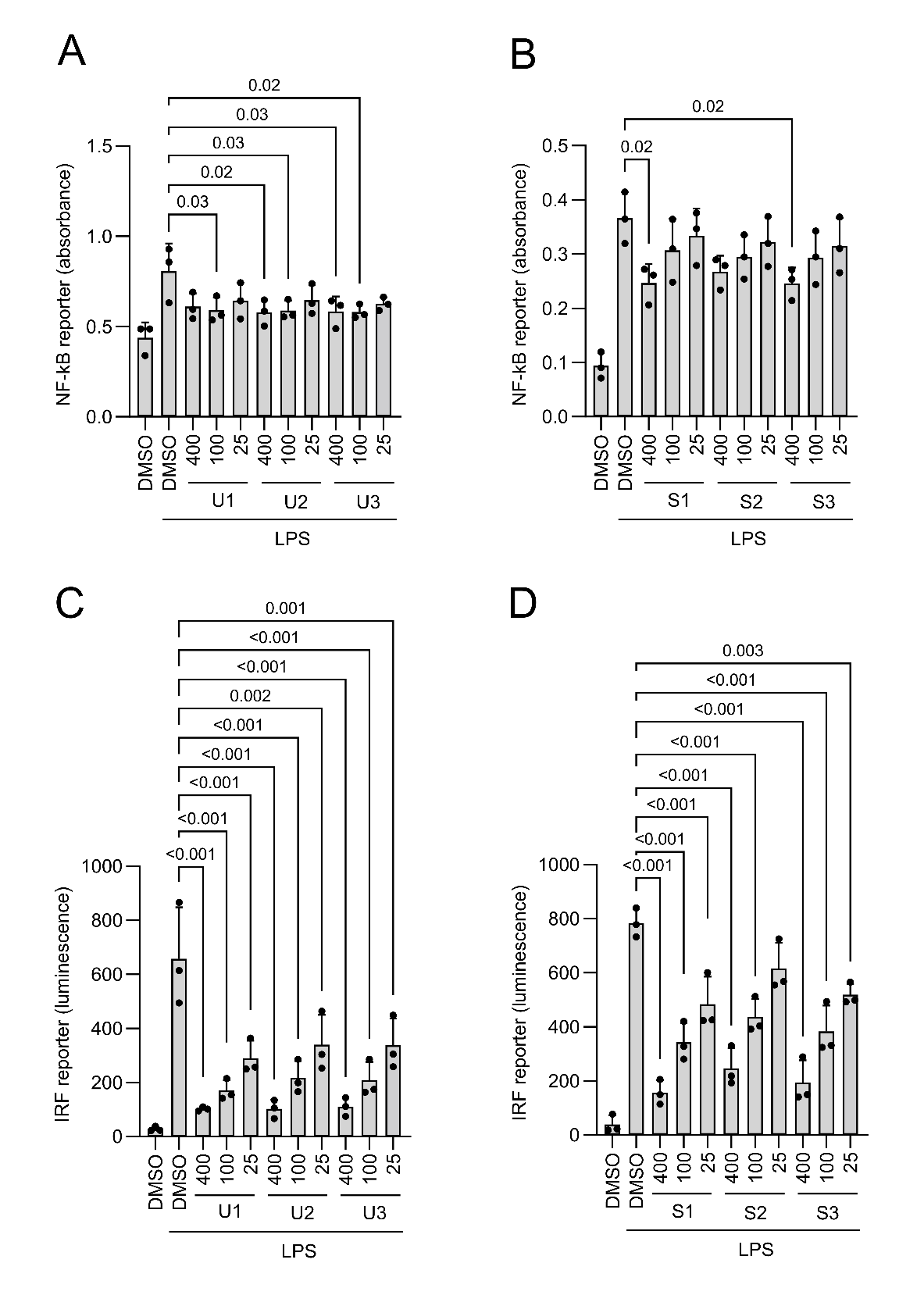
Supplementary information

##### Supplementary Figure 1

NF-κB reporter activation in THP-1 macrophages pretreated with *U. fenestrata,* indicated as U1-U3 (A) or *S. latissima,* indicated as S1-S3 (B) extracts in DMSO at indicated concentrations (400, 100 and 25 µg/ml) for 16 h, followed by LPS stimulation (10 ng/ml) for 24 h. IRF reporter activation in THP-1 macrophages pretreated with *U. fenestrata* (B) or *S. latissima* (D) extracts in DMSO at indicated concentrations (400, 100 and 25 µg/ml) for 16 h, followed by LPS stimulation (10 ng/ml) for 24 h. Shown are means with standard deviation and individual replicates. All treatments were compared to the LPS-stimulated control.

#####
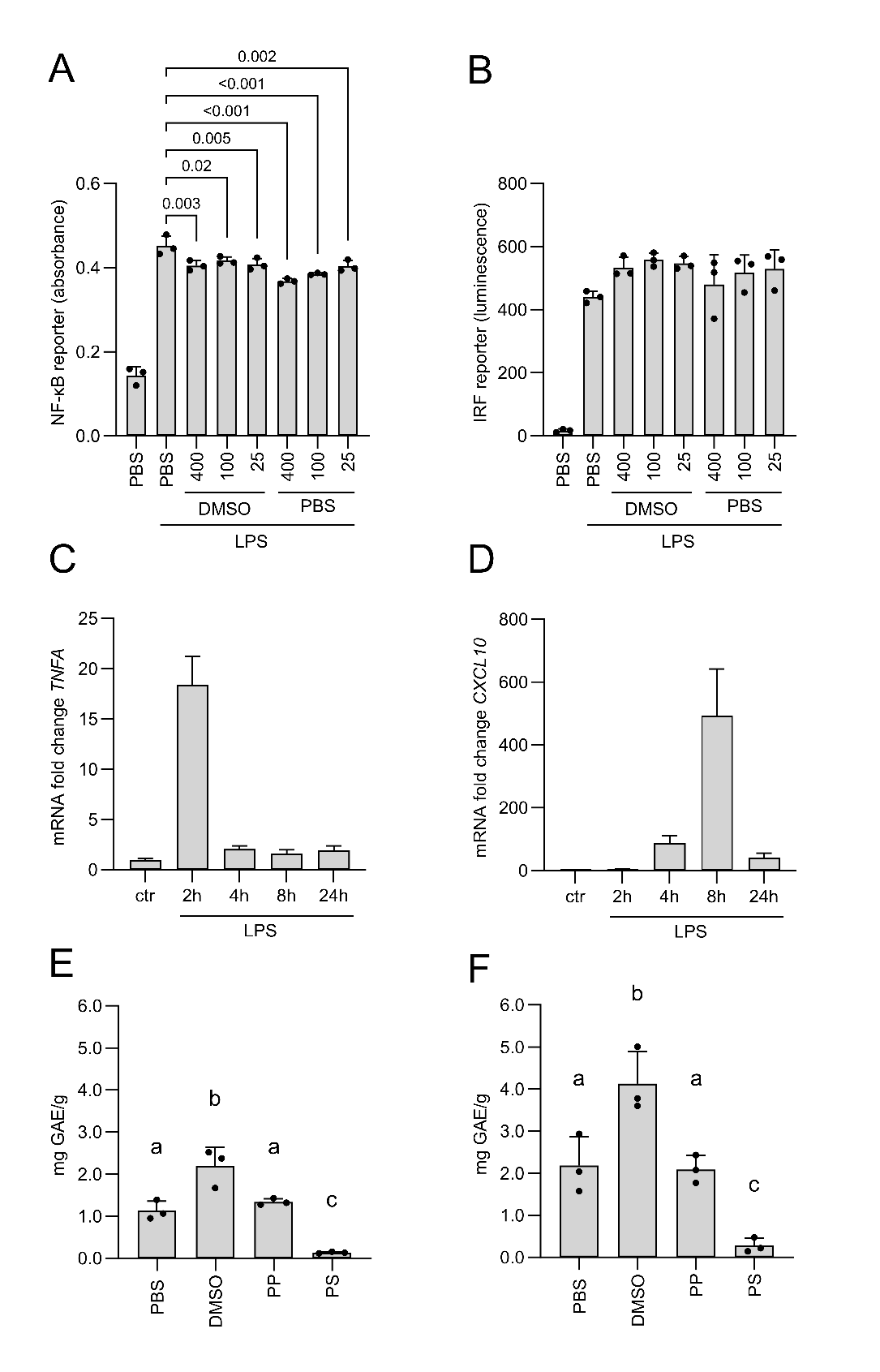


##### Supplementary Figure 2

THP1 macrophages were pretreated for 2 h with *S. latissima* (each replicate treated with either S1, S2 or S3) extracted in DMSO or PBS at the indicated concentrations (400, 100 and 25 µg/ml), then stimulated by LPS (10 ng/ml) for 24 h and NF-κB reporter (A) or IRF reporter (B) activation were measured. Shown are means with standard deviation and individual replicates. All treatments were compared to the LPS-stimulated control. Timeline of induction of *TNFA* (C) and *CXCL10* (D) by LPS stimulation (10 ng/ml) for indicated times in THP1 macrophages as assessed by qrt-PCR (n=1, technical duplicates). Total phenolic content as assessed by Folin-Ciocalteu assay in initial extracts as well as polyphenol (PP) and polysaccharide-enriched (PS) fractions from *U. fenestrata* (E) and *S. latissima* (F). All treatments were compared to each other, and similar means are indicated by the same superscript letter.
